# Genomic polymorphism of *Trifolium repens* root nodule symbionts from heavy metal-abundant 100-year-old waste heap in southern Poland

**DOI:** 10.1007/s00203-019-01708-x

**Published:** 2019-07-25

**Authors:** Ewa Oleńska, Wanda Małek

**Affiliations:** 1 Department of Genetics and Evolution, Institute of Biology, Faculty of Biology and Chemistry, University of Białystok, Ciołkowskiego 1J, 15-245 Białystok, Poland; 2 Department of Genetics and Microbiology, Institute of Microbiology and Biotechnology, Maria Curie Skłodowska University, Akademicka 19, 20-033 Lublin, Poland

**Keywords:** ERIC-PCR, Genomic diversity, Genomotyping, Heavy metals, REP-PCR, *Rhizobium leguminosarum* bv. *trifolii*

## Abstract

In total, 77 rhizobial strains isolated from the root nodules of *T. repens*, inhabiting heavy metal-contaminated waste heap (36 isolates) and control grassland (41 ones) in southern Poland, were analyzed for genome polymorphism and strength of the heavy metals’ (mainly Zn, Pb, Cd) selective pressure on bacterial genome polymorphism using two PCR-based techniques, ERIC- (enterobacterial repetitive intergenic consensus) and REP-PCR (repetitive extragenic palindromic) sequences. Both methods of different discriminatory power index (*D*) (ERIC-PCR *D *= 0.9737; REP-PCR *D *= 0.9826) allowed to distinguish 47 and 44 rhizobial strains, respectively. Combined analysis of ERIC-PCR and REP-PCR DNA amplicons differentiated all tested isolates. Both ERIC- and REP-PCR DNA fingerprinting techniques showed significant decline of the genome polymorphism (*h*) in rhizobial population from metalliferous waste heap (*h* = 0.89 ± 0.03; *h* = 0.90 ± 0.02, respectively) compared to rhizobia from control non-metalliferous area (*h* = 0.99 ± 0.01; *h* = 0.98 ± 0.02, respectively) as well as substantial differences in the genomic polymorphism between both these populations (*F*_ST_ = 0.162, *p *= 0.008; *F*_ST_ = 0.170, *p *= 0.000, respectively).

**Electronic supplementary material:**

The online version of this article (10.1007/s00203-019-01708-x) contains supplementary material, which is available to authorized users.

## Introduction

Rhizobia are a group of Gram-negative free-living soil bacteria that have enormous scientific and agronomic significance due to their ability to enter into the nitrogen-fixing symbiosis with legumes (*Fabaceae*), which is visible as nodules formed mainly on plant roots (Angelini et al. 2011). In nodules, rhizobia convert atmospheric diatomic nitrogen, by enzymatic complex dinitrogenase, into ammonium available for plants. Instead, a host plant supplies for rhizobia different nutrients, and creates them safe and favorable conditions for the growth and development. Such reciprocal benefits of both symbiotic partners increase their fitness and are crucial for plant and its microsymbionts existence, particularly in the wide range of different ecosystem types (Terefework et al. 2001).

The adaptation of natural bacterial populations to various habitats as well as to changing environmental conditions is determined genetically. It is an evolutionary process resulting from the accumulation of beneficial genes which control the adaptive phenotypes leading to the improvement of the bacterial population fitness (Olson-Manning et al. 2012). The level of the genetic variability is of fundamental importance in the adaptation of bacterial populations to environmental changes (Lynch 1996; Barret and Schluter 2008; Jump et al. 2008).

Natural populations of rhizobia usually exhibit the pronounced genetic diversity. It was noted that this group of soil microbes is sensitive to heavy metals, which usually are toxic for rhizobia even in small doses (Giller et al. 1998, 2009). In bacterial cells, heavy metals are capable of blocking essential functional groups of organic molecules, displacing essential metal ions, modifying the active conformations of biological molecules (Singh et al. 2011; Tchounwou et al. 2012), disturbing a respiratory electron transport chain, decreasing the efficiency of the substrate utilization resulting in a reduction of a bacterial growth, and inducing oxidative damages or genotoxicity (Chander et al. 2001, 2002; Alkorta et al. 2004; Lebeau et al. 2008) leading to the decrease in the total amount of soil microbial biomass (Barajas-Aceves 2005). It was found that noxious heavy metals may act as the natural selection factors changing the bacterial community structure (Abaye et al. 2005; Khan et al. 2010), reducing a genomic polymorphism, eliminating disadvantageous characteristics and favoring the metal-tolerant bacterial genotypes (Witter et al. 2000; Carrasco et al. 2005).

The selective properties of heavy metals and its deleterious impact on the genetic diversity of rhizobial populations were described in the case of few European populations inhabiting areas polluted mainly by zinc, lead, and cadmium deriving from the contaminated sewage sludge. Studies of *R. leguminosarum* bv. *trifolii* population from Estarreja (Portugal) showed a moderate heavy metals selective pressure on this bacterial community (Castro et al. 1997), whereas in the case of rhizobial population from Woburn (United Kingdom) an impressive decrease in the genetic variability was noted (Giller et al. 1998; Lakzian et al. 2002). The research in Braunschweig (Germany) showed the complete lack of *R. leguminosarum* bv. *trifolii* bacteria in heavy metal-polluted soil (Chaudri et al. 1993).

In Poland, the example of area with the high heavy metal concentration in soil is Olkusz Ore Region (OOR, Silesia-Cracow Uplands), where the post-industrial and post-mining deposits of zinc and lead ores were created artificially in the different period of time; some of them are derived from nineteenth century. One such, about 100 years old, waste heap is located in Bolesław (OOR). Enormously high heavy metals soil content, up to 40,000 mg Zn × kg^−1^, 1650 mg Pb × kg^−1^, and 170 mg Cd × kg^−1^ (Orłowska et al. 2002), deficiency in water and nutrients as well as the high insolation create highly disadvantageous conditions for microorganisms residing in Bolesław waste heap. Nevertheless, this calaminous area is inhabited by vegetation, which developed as a product of a natural succession process, and among other plant species, *Trifolium repens* together with its *Rhizobium leguminosarum* bv. *trifolii* microsymbionts are present in this area (Nowak et al. 2011; Oleńska and Małek 2013). The time required for the development of heavy metal resistance in plants varies from one to many decades (Antosiewicz 1992). It cannot be excluded that the age of Bolesław waste heap might be sufficiently long period to develop adaptations of plants and microbes to such unfavorable conditions and to observe microevolutionary processes occurring in this habitat.

The main purpose of the present study was to evaluate the level of the genomic diversity of *T. repens* nodule endosymbionts from metalliferous Bolesław waste heap and non-metalliferous control area and to determine the effect of heavy metals on a adaptability of rhizobial population to harmful conditions prevailing in about 70- to 100-year-old Zn−Pb waste heap using the ERIC- and REP-PCR DNA fingerprinting methods and to determine the role of heavy metals in rhizobium adaptability to heavy metal presence in the environment. Both these molecular techniques are based on the repetitive elements present in bacterium genomes, i.e., 127-bp enterobacterial repetitive intergenic consensus sequences (ERIC) found only within transcribed regions (ERIC-PCR) and repetitive extragenic palindromic elements of the length between 21 and 65 base pairs (bp) REP-PCR (Schneider and de Bruijn 1996; Stumpf et al. 2005).

## Materials and methods

### Bacterial strains

**Fig. 1:**
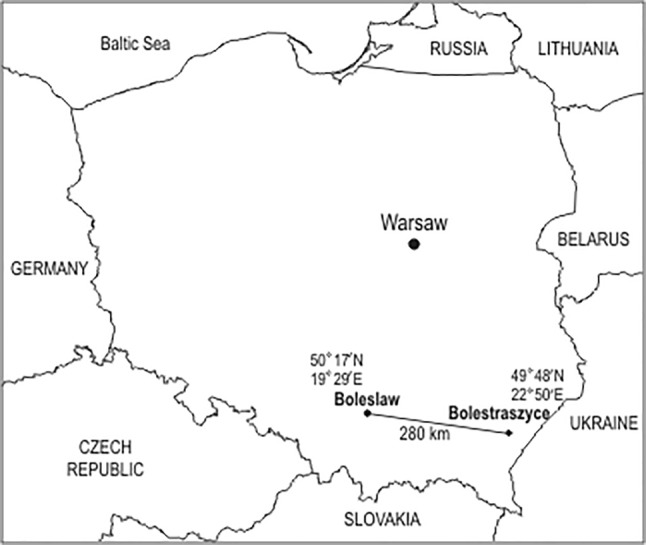
The location of areas and places on the map of Poland where the tested *T. repens* symbionts came from

The strains used in this study are listed in Table 1. Thirty six *Rhizobium leguminosarum* bv. *trifolii* isolates were obtained from the root nodules of *T. repens* growing in old (about 100 years old) calamine waste heap in Bolesław and 41 rhizobial isolates from root nodules of *T. repens* inhabiting a control grassland in Bolestraszyce (south Poland) (Fig. 1) with an average Zn, Pb, and Cd content of 50 mg, 21 mg and 0.30 mg per kg soil d. wt., respectively (Materna and Pęcek 2013). Root nodules surface sterilization, pure cultures isolation and also the plant nodulation test were performed according to a protocol described previously (Oleńska and Małek 2015). All bacteria were grown on the yeast extract-mannitol medium (YEM) at 28 °C and stored in the same medium at 4 °C (Vincent 1970).

**Table 1:** ERIC (A) and REP (B) genotypes, their frequencies (*f*), and strains of *R. leguminosarum* bv. *trifolii* received from nodules of *T. repens* growing in the Bolesław waste heap (metalliferous) (H), and control (non-metalliferous) area (K)

Number of genotypes	ERIC genotype	Bacterial strains	Frequency [*F*]	REP genotype	Bacterial strains	Frequency [*f*]
1	A1	1.3 K	0.013	B1	1.3 K	0.013
2	A2	1.4 K	0.013	B2	1.4 K, 6.3 K, 6.10 K	0.039
3	A3	1.6 K	0.013	B3	1.6 K, 1.7 K	0.026
4	A4	1.7 K	0.013	B4	1.8 K, 2.4 K, 2.5 K	0,039
5	A5	1.8 K	0.013	B5	2.1 K	0.013
6	A6	2.1 K	0.013	B6	2.7 K	0.013
7	A7	2.4 K, 3.2 K, 6.10 K	0.039	B7	2.9 K, 2.10 K, 3.2 K, 6.6 K, 6.9 K	0.065
8	A8	2.5 K	0.013	B8	3.3 K	0.013
9	A9	2.7 K	0.013	B9	3.5 K	0.013
10	A10	2.9 K, 6.6 K	0.026	B10	3.9 K	0.013
1	A11	2.10 K	0.013	B11	3.10 K	0.013
12	A12	3.3 K	0.013	B12	4.3 K	0.013
13	A13	3.5 K	0.013	B13	4.4 K, 4.8 K, 4.10 K	0.039
14	A14	3.9 K	0.013	B14	4.5 K, 4.7 K	0.026
15	A15	3.10 K	0.013	B15	5.3 K, 5.4 K	0.026
16	A16	4.3 K	0.013	B16	5.5 K, 5.6 K	0.026
17	A17	4.4 K	0.013	B17	5.7 K, 6.7 K, 1.9H	0.039
18	A18	4.5 K, 4.8 K, 4.10 K	0.039	B18	5.10 K	0.013
19	A19	4.7 K	0.013	B19	6.5 K	0.013
20	A20	5.3 K	0.013	B20	8.2 K, 8.8 K	0.026
21	A21	5.4 K	0.013	B21	8.3 K	0.013
22	A22	5.5 K	0.013	B22	9.2 K	0.013
23	A23	5.6 K	0.013	B23	9.3 K	0.013
24	A24	5.7 K	0.013	B24	9.7 K	0.013
25	A25	5.10 K	0.013	B25	9.9 K	0.013
26	A26	6.3 K	0.013	B26	1.1H	0.013
27	A27	6.5 K, 9.2 K	0.026	B27	1.2H, 1.4H	0.026
28	A28	6.7 K	0.013	B28	1.5H, 1.6H, 1.7H, 1.8H	0.052
29	A29	6.9 K	0.013	B29	1.10H	0.013
30	A30	8.2 K	0.013	B30	3.3H	0.013
31	A31	8.3 K	0.013	B31	3.5H	0.013
32	A32	8.8 K	0.013	B32	3.7H	0.013
33	A33	9.7 K	0.013	B33	4.1H, 4.2H	0.026
34	A34	9.3 K, 9.9 K	0.026	B34	4.3H	0.013
35	A35	1.1H, 1.2H, 1.4H, 1.5H, 1.6H, 1.7H, 1.8H, 1.9H, 1.10H	0.117	B35	4.4H	0.013
36	A36	3.3H, 3.5H	0.026	B36	4.4H, 4.51H	0.026
37	A37	3.7H	0.013	B37	5.1H	0.013
38	A38	4.1H	0.013	B38	5.2H, 5.5H	0.026
39	A39	4.2H	0.013	B39	5.4H	0.013
40	A40	4.3H,4.51H, 5.1H, 5.2H, 5.4H,5.5H	0.078	B40	6.3H, 6.5H, 6.12H, 6.13H	0.052
41	A41	4.4H	0.013	B41	7.1H, 7.2H, 7.3H	0.039
42	A42	4.5H	0.013	B42	7.4H, 7.6H, 7.7H	0.039
43	A43	6.3H, 6.5H, 6.12H, 6.13H	0.052	B43	8.2H, 8.5H	0.026
44	A44	7.1H, 7.2H	0.026	B44	8.3H, 8.1H	0.026
45	A45	7.3H, 7.4H, 7.6H, 7.7H	0.052			
46	A46	8.2H	0.013			
47	A47	8.3H, 8.1H, 8.5H	0.039

### DNA isolation

To isolate genomic DNA, the studied *R. leguminosarum* bv. *trifolii* strains were first cultured in 5 mL liquid YEM medium in an incubator shaker (150×*g*) for 18 h at 28 °C and next, they were transferred into 25 mL YEM broth, and again incubated in shaker for 72 h at 28 °C. Obtained bacterial cultures were centrifuged at 20,000×*g* for 15 min, the pellet was rinsed with 0.5 M NaCl for removal of the exopolysaccharides and further centrifuged at 20,000×g for 5 min. Finally, the received bacterial pellet was suspended in the Tris buffer and subjected to a total genomic DNA extraction following a standard protocol supplied with Genomic Mini kit (A&A Biotechnology). The purity and the concentration of DNA were measured using nanodrop (Thermo Scientific). DNA concentration was adjusted to 100 ng/μL.

### Strain genotyping by ERIC-PCR

For fingerprinting of *R. leguminosarum* bv. *trifolii* strain genomes by ERIC-PCR method, each DNA amplification reaction was set up in 5 µL mixture containing 1.650 µL of Multiplex PCR Master mix (Quiagen), 1.0 µL of RNase-free water (Quiagen), 0.350 µL of primer mixture (0.3 µM of each primer) and 2 µL of DNA (100 ng/μL). The ERIC-PCR DNA amplification with primers ERIC-1R (5′ ATGTAAGCTCCTGGGGATTCAC-3′) and ERIC-2 (5′-AAGTAAGTGACTGGGGTGAGCG-3′) (Versalovic et al. 1991) was carried out in TProfessional thermocycler (Biometra) with the following temperature profile: initial denaturation at 95 °C for 15 min, 30 cycles of denaturation at 90 °C for 30 s, annealing at 58 °C for 1 min, extension at 65 °C for 8 min, and final extension at 68 °C for 16 min (Versalovic et al. 1998).

### Strain genotyping by REP-PCR

For the determination of a genomic diversity of the studied rhizobia, the REP-PCR was performed in 15 μL mixture consisting of 4.950 μL of Multiplex PCR Master mix (Quiagen), 3.0 µL of RNase-free water (Quiagen), 1.050 µL of primer mixture (0.3 µM of each primer) and 6 µL of DNA (100 ng/μL). The DNA fragments present between genomic dispersed repetitive extragenic palindromic elements were amplified with primers REP2-I (5′-ICGICTTATCIGGCCTAC-3′) and REP1R-I (5′-IIIICGICGICATCIGGC-3′) (Versalovic et al. 1991; de Bruijn 1992) in conditions as follows: initial denaturation at 95 °C for 15 min, 40 cycles of denaturation at 94 °C for 1 min, annealing at 40 °C for 1 min, extension at 65 °C for 8 min and final extension at 65 °C for 16 min (Versalovic et al. 1998).

### Analysis of the ERIC-PCR and REP-PCR products

The amplified DNA fragments from ERIC- and REP-PCR methods were separated and identified in 1.5% agarose gels in 1 × TBE buffer (0.1 M Tris, 0.1 M boric acid, 0.2 mM EDTA) for 2 h at 80 V. All DNA gel patterns, stained with ethidium bromide, were analyzed under UV light in a gel documentation system with ChemiDoc XRS + System (Bio-Rad Laboratories, Hercules, USA) and photographed (Bio-Rad Laboratories, Hercules, USA apparatus). The sizes of ERIC- and REP-PCR DNA products were assigned according to molecular weight markers: GeneRuler™ 100 bp DNA Ladder SM0241 100−1000 bp, Fast Ruler™ Low Range DNA Ladder SM1103 50−1500 bp, and Fast Ruler™ Middle Range DNA Ladder SM1113 100−5000 bp (Fermentas).

### Data analysis

The obtained ERIC-PCR and REP-PCR genome patterns were converted to the binary matrix (1—presence, 0—absence of band of a specific molecular weight) with the usage of FenAl 1.0β software. Strain similarities were determined by a simple matching coefficient, next clustering correlation coefficients were calculated by the unweighted pair group method with arithmetic averages (UPGMA) and phenograms were generated by SAHN algorithm (sequential agglomerative hierarchical and nested) of NTSYS-pc 2.02 g analysis package (Applied Biostatistics Inc.) (Rohlf 2009).

For the estimation the discriminatory power of ERIC- and REP-PCR techniques and detection a more effective method for strains diversification, the numerical indices of the discriminatory abilities of ERIC- and REP-PCR methods were calculated by applying Simpson’s index of diversity (*D*), which equation is given as follows:

$$D = 1 - \frac{1}{{N\left( {N - 1} \right)}}\mathop \sum \limits_{i = 1}^{S} n_{i} \left( {n_{i} - 1} \right),$$

where *N* is the total number of strains in the sample population, *S* is the total number of genotypes described, and *n*_*i*_ is the number of isolates belonging to the *i*’th genotype (Nath et al. 2010).

To determine the level of population strain richness and the index of strain diversity (Fernández-Pérez et al. 2010), measured as the frequency of diverse genotypes, the following equation was used:

$${\text{ISD}} \left[ \% \right] = 100{\text{\% }}\frac{\text{number of genotypes}}{\text{total number of isolates}}$$

To determine a relative genotype richness of bacterial populations from both studied areas, established based on the genome DNA patterns obtained by ERIC- and REP-PCR techniques, the popular Shannon's diversity index (*H’*) was used according to the formula:

$$H^{\prime} = - \mathop \sum \limits_{i = 1}^{S} p_{i} { \ln }p_{i} ,$$

where *p*_*i*_ is a relative abundance of each genotype calculated as the proportion of number of isolates representing the specific genotype to the total number of isolates and *S* is a total number of isolates belonging to *i'*th genotype (Seguin et al. 2001; Farooq and Vessey 2009). For the examination the significance of the differences in genotype richness between both rhizobial populations, their Shannon’s diversity index (*H’*) values were subjected to the non-parametric *U* Mann–Whitney statistical test at the significance level of 0.05 with the usage of Statistica 13 program.

The level of the intrapopulation genomic diversity was measured with the usage of the standard genotype diversity index (*h*) (Hartl and Clark 2007). The differences in the genotype frequencies (*f*) between metalliferous and control bacterial populations were determined by the fixation index (*F*_ST_) (Neigel 2002; Pearse and Crandall 2004) and presented as the minimum spanning trees (MSTs) constructed with the usage of Arlequin 3.5.1.2 software (Excoffier and Lischer 2010). The MST presents the network of relatedness of the studied genotypes, represented by nodes connected by branches of different length, illustrating the genetic distances between the genotypes (Spada et al. 2004).

## Results

### ERIC and REP-PCR profile analysis

The analysis of the genomic polymorphism of 77 rhizobial strains from the root nodules of *T. repens*, growing in a heavy metal contaminated waste heap (Bolesław) and control non-metalliferous area (Bolestraszyce), showed the high, comparable diversification powers of both ERIC-PCR (*D *= 0.9737) and REP-PCR (*D *= 0.9826) genotyping methods (Table S1). The usage of ERIC-PCR technique allowed to differentiate 47 genotypes (ISD = 61%), while REP-PCR generated 44 ones (ISD = 57%) among 77 rhizobia studied. 75% rhizobial genotypes generated by ERIC-PCR method were represented by only a single isolate, including 47% rhizobial genotypes from waste heap and 85% ones from the control area, whereas among all bacterial genotypes identified by REP primers 55% of them were represented by only a single isolate including 60% genotypes from Bolestraszyce population, and 47% ones representing rhizobia from the Bolesław waste heap. Among genotypes identified by ERIC-PCR technique, the most frequent genotype in a waste heap rhizobial population was a genotype A35 (*f *= 0.12) represented by nine strains and the genotypes A7 and A18 detected in a control population represented by three isolates with a frequency *f *= 0.039 (Table 1). Among genotypes identified by REP primers in a control population, the most frequent was genotype B7 (*f *= 0.065). In the metalliferous Bolesław population, the highest frequency, among REP-PCR created genotypes, showed genotypes B28 and B40 (*f *= 0.052). ERIC-PCR generated DNA profiles contained 2−11 bands (average 5.5 amplicons per isolate), ranging in size from 0.150 to 5.000 kbp, whereas REP-PCR products consisted of 1−12 DNA fragments (average 5.5 amplicons per isolate) of 0.200−5.000 kbp length. In ERIC- and REP-PCR methods, the DNA patterns of rhizobia from the waste heap as well as control source were similar in the number and the size range.

### Genotyping diversity of both studied rhizobial populations

Analysis of DNA profiles of 77 *T. repens* root nodule isolates obtained by ERIC- and REP-PCR methods revealed differences in the number various genotypes in both metalliferous and non-metalliferous rhizobial populations expressed by Shannon’s diversity index (*H’*) values. *H’* index achieved the higher value in the case of the control rhizobial population (3.0734, 3.4212) than in metalliferous one (2.6547, 2.2632) by REP- and ERIC-PCR methods, respectively (Table S1). Noted differences in *H’* values between both studied populations were statistically significant (*p *= 0.005, ERIC-PCR, and *p *= 0.000, REP-PCR). In a consequence, the significant reduction of the genomic diversity of the rhizobial population from metalliferous Bolesław area in comparison with the Bolestraszyce control one was found. Based on ERIC- and REP-PCR genome fingerprinting techniques, significantly lower level of the genotype diversity (*h*) was detected in the waste heap Bolesław population (0.89 ± 0.03, 0.90 ± 0.02) than in a control Bolestraszyce one (0.99 ± 0.01, 0.98 ± 0.02), respectively. Moreover, significant variances in genotype frequencies between metalliferous and non-metalliferous rhizobial populations were also supported by the fixation index *F*_ST_ which achieved the value of 0.162 (*p *= 0.008) in the case of ERIC-PCR, and 0.170 (*p *= 0.000) in REP-PCR methods (Table S1). According to Hartl and Clark (2007), these *F*_ST_ values may indicate a high level of the genomic differences between both studied *T. repens* endosymbionts populations (Table S1). Regarding the similar number of analyzed isolates deriving from heavy metal contaminated (36 isolates), and non-contaminated (41 isolates) areas, respectively, significantly less genotypes were identified in *R. leguminosarum* bv. *trifolii* waste heap population (13 by ERIC-PCR, 19 by REP-PCR) in comparison to Bolestraszyce control one (34 by ERIC-PCR, 25 by REP-PCR). It suggests that *R. leguminosarum* bv. *trifolii* bacteria have adapted to the heavy metal presence in the environment and that these harmful agents select genotypes adjusted to them.

**Fig. 2:**
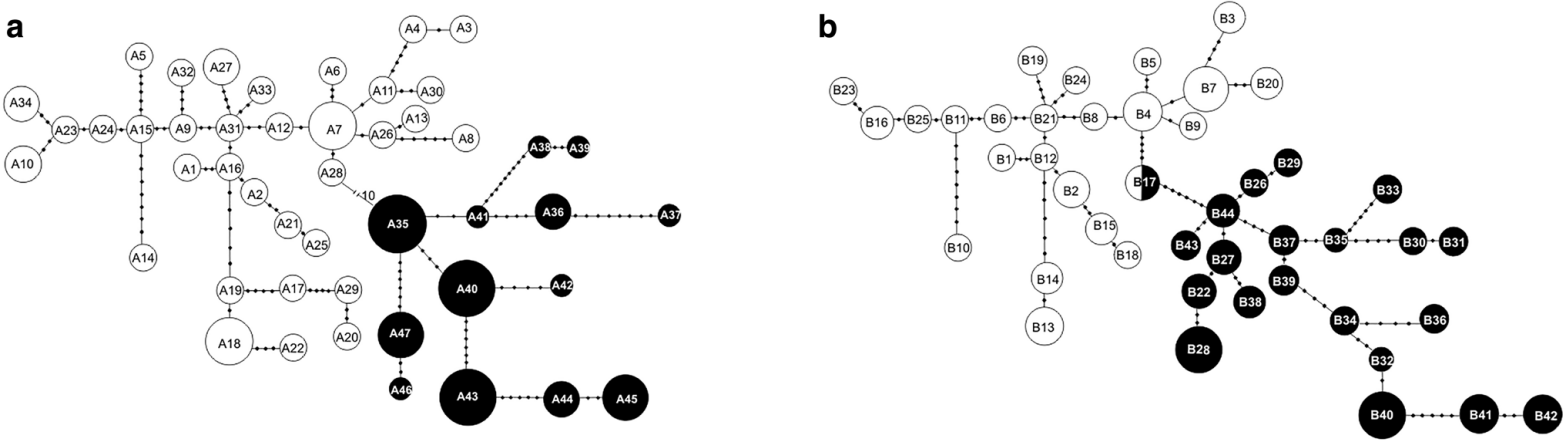
Minimum spanning trees (MSTs) presenting the network of relatedness of *R. leguminosarum* bv. *trifolii* genotypes identified in the bacterial populations from the Bolesław waste heap and Bolestraszyce control area by ERIC- (**a**) and REP-PCR (**b**) DNA fingerprinting methods (symbols A1−A47 for genotypes from ERIC-PCR method; symbols B−B44 for genotypes from REP-PCR method). The size of the circles (nodes) corresponds to the genotype frequency in the given population. The number of small dots on the tree edges corresponds to the differences in the number of various DNA bands between *R. leguminosarum* bv. *trifolii* genotypes (A1−A47, and B−B44). Number 10 corresponds to the amount of distinct DNA bands between the genotypes A28 and A35

The genomic polymorphism and genomic relationship of *R. leguminosarum* bv. *trifolii* strains from metalliferous Bolesław and non-metalliferous Bolestraszyce areas were graphically visualized by the minimum spanning trees (MSTs) (Fig. 2). The genotypes A7 and A31 of *R. leguminosarum* bv. *trifolii,* identified by ERIC-PCR method in the control non-metalliferous population, are presented on a dendrogram as nodes with the highest number of edges, which allows to think that they are the most possible ancestral genotypes of other ones. Among genotypes identified by ERIC-PCR in the rhizobial population from a Bolesław waste heap, the genotypes A35, A40, and A41 are the potential ancestors of other genotypes from metalliferous area (Fig. 2a). The minimum spanning tree presenting the network interrelatedness between the genotypes identified by REP-PCR shows that possible ancestors of other white clover symbionts from a control grassland are the B4 and B21 genotypes, whereas in a waste heap rhizobial population the genotype B44 is a presumable ancestor of other genotypes identified in this metalliferous heap (Fig. 2b).

In the UPGMA cluster analysis of ERIC-PCR DNA fingerprints, all 77 *R. leguminosarum* bv. *trifolii* isolates were grouped together on dendrogram at the coefficient identity of 0.64, and formed four main bacterial clusters and four separate branches at the coefficient identity of 0.76. The determined bacterial clusters were homogeneous regarding the origin of isolates; two of them were composed of rhizobia deriving from Bolesław waste heap, the other two comprised symbionts of *T. repens* from Bolestraszyce field. The amplification reaction with ERIC primer set generated the unique strain-specific DNA profiles for 35 rhizobia (45.5%) allowing for differentiating them from all other ones. The remaining root nodule isolates formed on UPGMA dendrogram sub-groups consisting of 2, 3, 4, 5, 6, and 9 strains with an identical DNA profiles (Fig. S1).

For molecular typing and analysis of genomic polymorphism of all studied rhizobia deriving from metalliferous Bolesław and non-metalliferous Bolestraszyce areas, PCR technique based on REP primers has been also used. The UPGMA dendrogram, derived from REP-PCR DNA fingerprinting data, showed that nodule bacteria, at the coefficient identity of 0.71, were grouped together into two major sub-clusters, nonhomogeneous in terms of strain origin, and formed four independent lineages on the outskirt of the tree (Fig. S2). REP-PCR technique did not also allow to distinguish all strains one from another. 24 strains studied (31.2%) exhibited the unique DNA patterns for individual bacteria. The remaining rhizobia formed on dendrogram based on REP-PCR DNA fingerprinting sub-groups with 2, 3, 4, and 5 different strains. Our results showed that both ERIC- and REP-PCR DNA fingerprinting methods have a similar discriminatory power and enabled to differentiate 47 and 44 genotypes, respectively.

**Fig. 3:**
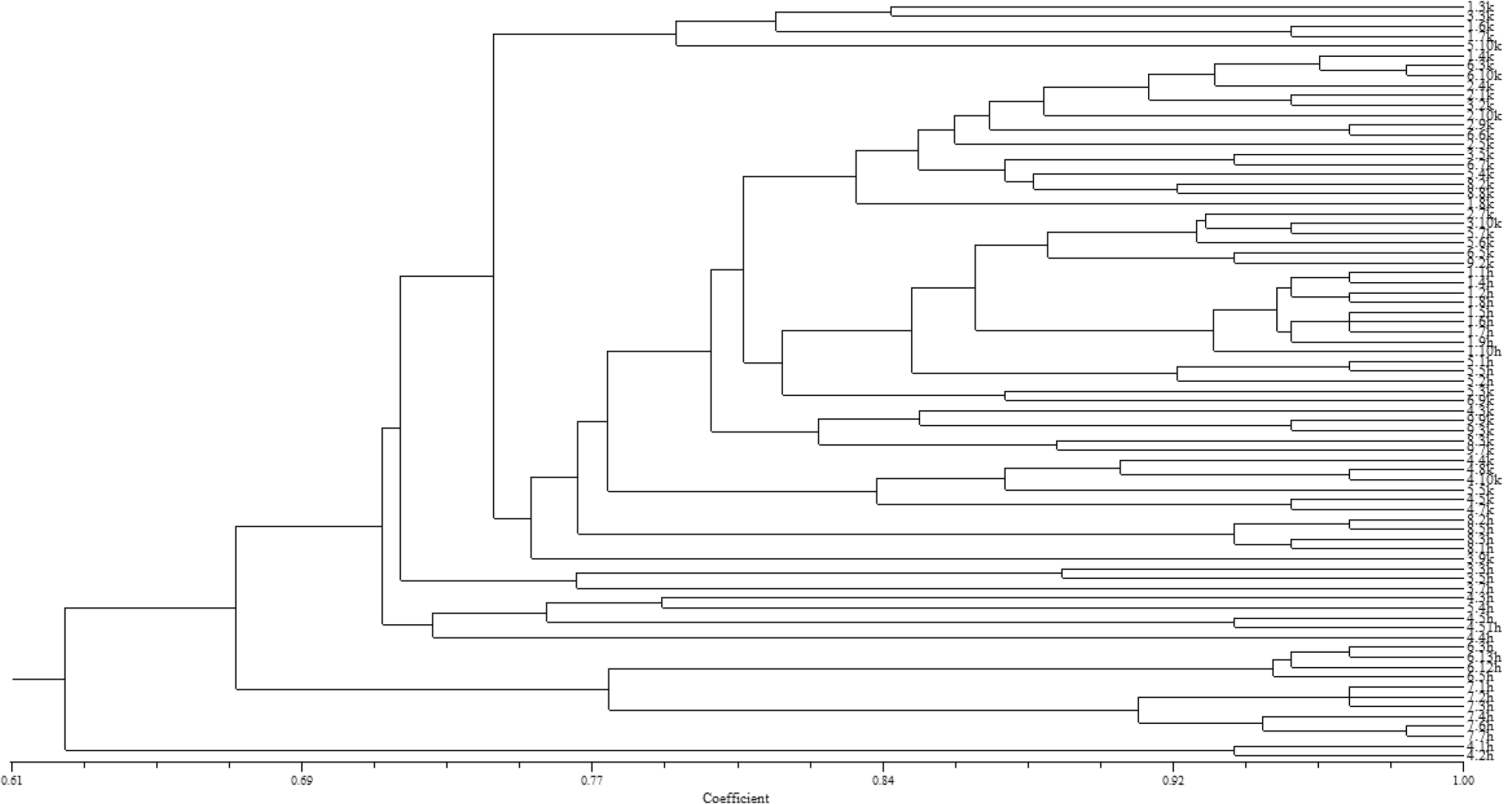
Dendrogram showing the genome diversity of *Trifolium repens* nodule microsymbionts obtained from Bolesław waste heap (H), and Bolestraszyce control area (K), based on the combined ERIC- and REP-PCR DNA patterns. Cluster analysis was performed by the UPGMA method. The scale at the top of the dendrogram presents the bacterial genome similarity rate (%) of 77 studied strains

The concatenated analysis of DNA patterns obtained by the ERIC- and REP-PCR methods allowed to differentiate all 77 analyzed symbionts of *T. repens* (*D *= 1.0) from metalliferous and non-metalliferous areas (Fig. 3).

## Discussion

DNA fingerprinting techniques such as ERIC-PCR based on enterobacterial repetitive intergenic consensus sequences (ERIC) and REP-PCR based on repetitive extragenic palindromic (REP) elements have been evaluated as highly useful methods in the bacterial strains identification and differentiation as well as in the analysis of bacterial genome polymorphisms (Carson et al. 2003; Stumpf et al. 2005; Gnat et al. 2015). Repetitive genomic sequences, such as ERIC and REP, containing highly conserved palindromic inverted repeats are widely dispersed in bacterial genomes and have been used as a tool for the molecular typing and differentiation the bacterial strains of various genera, i.e., *Mesorhizobium* sp., *Bacillus* sp., *Bordetella* sp., *Bartonella* sp., *Eschericha* sp., *Listeria* sp., *Mycobacterium* sp., *Salmonella* sp. (Ferreira et al. 2001; Sampaio et al. 2006; Yang et al. 2009; Gnat et al. 2015). ERIC elements are 127-bp sequences which consist of highly conserved inverted repeats located in the intergenic regions whereas REP sequences are 21−65-bp palindromic units, which contain a 5-bp variable loop and are located in extragenic regions of DNA (Versalovic et al. 1998; Lindsay and Sharp 2006). Although the function of both these molecular markers is unknown, it is assumed that they are involved in a homologous recombination, enhancement of the expression of the flanking genes due to providing binding sites for proteins and enzymes, and increase the longevity of mRNA of genes (de Bruijn 1992; Wilson and Sharp 2006). ERIC and REP elements are separated by various length DNA fragments, which are amplified in polymerase chain reaction with ERIC and REP primers and presented as different size DNA amplicons characteristic for individual bacterial strains (Saxena et al. 2002). Many papers revealed that the above-mentioned genome fingerprinting techniques may be highly useful in the analysis of the genomic polymorphism of different rhizobial populations (de Bruijn 1992; Laguerre et al. 1996; Farooq and Vessey 2009; Gnat et al. 2015).

In the previous paper, Oleńska and Małek (2015) showed statistically essential decline of the genetic diversity of white clover symbionts from the metalliferous Bolesław waste heap compared to rhizobial population from non-metalliferous Bolestraszyce control area by *nifH* (nitrogenase) gene sequence analysis despite the fact that all identified *nifH* gene mutations were silent and did not affect the primary structure of nitrogenase reductase (NifH). In this study to determine the level of genomic polymorphism and the strength of heavy metal selective pressure on the genome polymorphism of *T. repens* symbionts, 36 rhizobial strains from metalliferous Bolesław and 41 ones from non-metalliferous Bolestraszyce areas (Table 1) were studied by ERIC- and REP-PCR DNA fingerprinting techniques. The both used methods turned out to be highly useful in a diversification of studied rhizobia. ERIC-PCR enabled to differentiate 61% of strains (*D *= 0.9737) and REP-PCR almost 57% of strains among 77 bacteria studied (*D *= 0.9826) (Table S1). The genetic distances between concatenated genotypes of rhizobia from Bolesław waste heap and control non-metalliferous area determined on the basis of ERIC- and REP-PCR DNA patterns were presented by the network analysis (Fig. 2). Both these trees showed the distant genomic relationship between rhizobial genotypes from metalliferous and non-metalliferous areas, with the exception of the B17 genotype which was shared by both studied bacterial populations, as well as showed close relationship of rhizobial genotypes within each population.

Neither ERIC-PCR nor REP-PCR methods have enabled to differentiate all studied bacteria from each other. Unique genotypes were attributed to each rhizobial strain by combined analysis of ERIC- and REP-PCR DNA patterns (*D *= 1.0) illustrating that two used DNA fingerprinting techniques are highly useful in determination of the valid level of the genomic diversity of heavy metal influenced *R. leguminosarum* bv. *trifolii* population and white clover symbionts from the control non-metalliferous area (Fig. 3).

Suggested in this paper role of the heavy metals in the selection of *R. leguminosarum* bv. *trifolii* genotypes, adapted to these harmful metals, has also been noticed earlier (Chaudhary et al. 2004; Wang et al. 2007). Heavy metals, mainly Zn, Pb or Cd, have also revealed their genotype-specific selective properties with respect to other rhizobia, e.g., *Rhizobium meliloti* (Carrasco et al. 2005), *Bradyrhizobium* sp. (Wani et al. 2007). Despite the evident decline of the genomic diversity of studied rhizobia under the heavy metal stress (*h *= 0.89 ± 0.03 ERIC; *h *= 0.90 ± 0.02 REP) compared to a non-metalliferous rhizobial population (*h *= 0.99 ± 0.01 ERIC; *h *= 0.98 ± 0.02 REP), as well the substantial genomic differentiation between both studied populations (*F*_ST_ = 0.162, *p *= 0.008 ERIC; *F*_ST_ = 0.170, *p *= 0.000 REP) (Table S1), the level of this reduction was not so impressive as in other European populations inhabiting long-term contaminated wastes (Chaudri et al. 1993; Castro et al. 1997; Giller et al. 1998). However, it can be concluded that the studied *R. leguminosarum* bv. *trifolii* population from Bolesław metalliferous waste heap exhibits sufficient adaptive potential to harmful heavy metals present there.

## Electronic supplementary material

Below is the link to the electronic supplementary material.

Supplementary material 1 (DOC 27 kb)

Supplementary material 2 (DOC 27 kb)

Supplementary material 3 (DOCX 14 kb)

## References

[CR1] Abaye DA, Lawlor K, Hirsch PR, Brookes PC (2005). Changes in the microbial community of an arable soil caused by long-term metal contamination. Europ J Soil Sci.

[CR2] Alkorta I, Hernández-Allica J, Becerril JM, Amezaga I, Albizu I, Garbisu C (2004). Recent findings on the phytoremediation of soils contaminated with environmentally toxic heavy metals and metalloids such as zinc, cadmium, lead, and arsenic. Rev Environ Sci Biotech.

[CR3] Angelini J, Ibáñez F, Taurian T, Tonelli ML, Valetti L, Fabra A (2011). A study on the prevalence of bacteria that occupy nodules within single peanut plants. Curr Microbiol.

[CR4] Antosiewicz DM (1992). Adaptation of plants to an environment polluted with heavy metals. Acta Soc Bot Polon.

[CR5] Barajas-Aceves M (2005). Comparison of different microbial biomass and activity measurement methods in metal-contaminated soils. Biores Technol.

[CR6] Barret RDH, Schluter D (2008). Adaptation from standing genetic variation. Trends Ecol Evol.

[CR7] Carrasco JA, Armario P, Pajuelo E, Burgos A, Caviedes MA, López R, Chambera MA, Palomares AJ (2005). Isolation and characterisation of symbiotically effective *Rhizobium* resistant to arsenic and heavy metals after the toxic spill at the Aznalcóllar pyrite mine. Soil Biol Biochem.

[CR8] Carson C, Carson A, Shear BL, Ellersieck MR, Schnell JD (2003). Comparison of ribotyping and repetitive extragenic palindromic-PCR for identification of fecal *Escherichia coli* from humans and animals. Appl Environ Microbiol.

[CR9] Castro IV, Ferreira EM, McGrath SP (1997). Effectiveness and genetic diversity of *Rhizobium leguminosarum* bv. *trifolii* isolates in Portuguese soils polluted by industrial effluents. Soil Biol Biochem.

[CR10] Chander K, Dyckmans J, Joergensen R, Meyer B, Raubuch M (2001). Different sources of heavy metals and their long-term effects on soil microbial properties. Biol Fertil Soils.

[CR11] Chander K, Dyckmans J, Hoeper H, Joergensen RG, Raubuch M (2002). Long-term effects on soil microbial properties of heavy metals from industrial exhaust deposition. J Plant Nutr Soil Sci.

[CR12] Chaudhary P, Dudeja SS, Kapoor KK (2004). Effectivity of host-*Rhizobium leguminosarum* symbiosis in soils receiving sewage water containing heavy metals. Microbiol Res.

[CR13] Chaudri AM, McGrath SP, Giller KE, Rietz E, Sauerbeck D (1993). Enumeration of indigenous *Rhizobium leguminosarum* bv. *trifolii* in soils previously treated with metal-contaminated sewage sludge. Soil Biol Biochem.

[CR14] de Bruijn FJ (1992). Use of repetitive (repetitive extragenic palindromic and enterobacterial repetitive intergeneric consensus) sequences and the polymerase chain reaction to fingerprint the genomes of *Rhizobium meliloti* isolates and other soil bacteria. Appl Environ Microbiol.

[CR15] Excoffier L, Lischer HEL (2010). Arlequin suite ver 3.5: a new series of programs to perform population genetics analyses under Linux and Windows. Mol Ecol Res.

[CR16] Farooq FT, Vessey JK (2009). Genetic diversity of *Bradyrhizobium japonicum* with soybean growing regions of the north-eastern Great Plains of North America as determined by REP-PCR and ERIC-PCR profiling. Symbiosis.

[CR17] Fernández-Pérez R, Torres C, Sanz S, Ruiz-Larrea F (2010). Strain typing of acetic acid bacteria responsible for vinegar production by the submerged elaboration method. Food Microbiol.

[CR18] Ferreira AMT, Suzart S, Vidotto O, Knowles DP, Vidotto MC (2001). Use of repetitive DNA elements to define genetic relationships among *Anaplasma marginale* isolates. FEMS Microbiol Lett.

[CR19] Giller KE, Witter E, McGrath SP (1998). Toxicity of heavy metals to microorganisms and microbial processes in agricultural soils: a review. Soil Biol Biochem.

[CR20] Giller KE, Witter E, McGrath SP (2009). Heavy metals and soil microbes. Soil Biol Biochem.

[CR21] Gnat S, Małek W, Oleńska E, Trościańczyk A, Wdowiak-Wróbel S, Kalita M, Wójcik M (2015). Insight into the genomic diversity and relationship of *Astragalus glycyphyllos* symbionts by RAPD, ERIC-PCR, and AFLP fingerprinting. J Appl Genetics.

[CR22] Hartl DL, Clark AG (2007). Principles of population genetics.

[CR23] Jump AS, Marchant R, Peñuelas J (2008). Environmental change and the option value of genetic diversity. Trends Plant Sci.

[CR24] Khan S, Hesham AE-L, Qiao M, Rehman S, He J-Z (2010). Effects of Cd and Pb on soil microbial community structure and activities. Environ Sci Pollut Res.

[CR25] Laguerre G, Mavingui P, Allard MR, Charnay MP, Louvrier P, Mazurier SI, Rigottier-Gois L, Amarger N (1996). Typing of rhizobia by PCR DNA fingerprinting and PCR-restriction Fragment length polymorphism analysis of chromosomal and symbiotic gene regions: application to *Rhizobium leguminosarum* and its different biovars. Appl Environ Microbiol.

[CR26] Lakzian A, Murphy P, Turner A, Beynon JL, Giller KE (2002). *Rhizobium leguminosarum* bv. *viciae* populations in soils with increasing heavy metal contamination: abundance, plasmid profiles, diversity and metal tolerance. Soil Biol Biochem.

[CR27] Lebeau T, Braud A, Jezequel K (2008). Performance of bioaugmentation-assisted phytoextraction applied to metal contaminated soils: a review. Environ Pollut.

[CR28] Lindsay AW, Sharp MP (2006). Enterobacterial repetitive intergenic consensus (ERIC) sequences in *Escherichia coli*: evolution and implications for ERIC-PCR. Mol Biol Evol.

[CR29] Lynch M, Avise JC, Hamrick JL (1996). A quantitative-genetic perspective on conservation issues. In:. Conservation genetics: case histories from nature.

[CR30] Materna G, Pęcek J, Lipińska EJ (2013). The quality of agricultural soils. Report on the state of the environment in Podkarpackie Voivodeship in 2012.

[CR31] Nath G, Maurya P, Gulati AK (2010). ERIC PCR and RAPD based fingerprinting of *Salmonella* Typhi strains isolated over a period two decades. Inf Genet Evol.

[CR32] Neigel JE (2002). Is FST obsolete?. Conservation Genet.

[CR33] Nowak T, Kapusta P, Jędrzejczyk-Korycińska M, Szarek-Łukaszewska G, Godzik B (2011). The vascular plants of the Olkusz Ore-bearing region.

[CR34] Oleńska E, Małek W (2013). Sequence analysis of hypothetical lysine exporter genes of *Rhizobium leguminosarum* bv. *trifolii* from calamine old waste heaps and their evolutionary history. Curr Microbiol.

[CR35] Oleńska E, Małek W (2015). Genetic differentiation of *Trifolium repens* microsymbionts deriving from Zn−Pb waste-heap and control area in Poland. J Basic Microbiol.

[CR36] Olson-Manning CF, Wagner MR, Mitchell-Olds T (2012). Adaptive evolution: evaluating empirical support for theoretical predictions. Nat Rev Genet.

[CR37] Orłowska E, Zubek Sz, Jurkiewicz A, Szarek-Łukaszewska G, Turnau K (2002). Influence of restoration on arbuscular mycorrhiza of *Biscutella laevigata* L. (Brassicaceae) and *Plantago lanceolata* L. (Plantaginaceae) from calamine spoil mounds. Mycorrhiza.

[CR38] Pearse DE, Crandall KA (2004). Beyond FST: analysis of population genetic data for conservation. Conservation Genet.

[CR39] Rohlf FJ (2009). NTSYS-pc: Numerical Taxonomy and Multivariate Analysis System, version 2.2.

[CR40] Sampaio JLM, Viana-Niero C, de Freitas D, Höfling-Lima AL, Cardoso Leão S (2006). Enterobacterial repetitive intergenic consensus PCR is a useful tool for typing *Mycobacterium chelonae* and *Mycobacterium abscessus* isolates. Diagn Microbiol Inf Dis.

[CR41] Saxena MK, Singh VP, Lakhcharua BD, Taj G, Sharma B (2002). Strain differentiation of Indian isolates of *Salmonella* by ERIC-PCR. Res Vet Sci.

[CR42] Schneider M, de Bruijn FJ (1996). Rep-PCR mediated genomic fingerprinting of rhizobia and computer-assisted phylogenetic pattern analysis. World J Microbiol Biotechnol.

[CR43] Seguin P, Graham PH, Sheaffer CC, Ehlke NJ, Russelle MP (2001). Genetic diversity of rhizobia nodulating *Trifolium ambiguum* in North America. Can J Microbiol.

[CR44] Singh R, Gautam N, Mishra A, Gupta R (2011). Heavy metals and living systems: an overview. Ind J Pharmacol.

[CR45] Spada E, Sagliocca L, Sourdis J, Garbuglia AR, Poggi V, De Fusco C, Mele A (2004). Use of the minimum spanning tree model for molecular epidemiological investigation of a nosocomial outbreak of hepatitis C virus infection. J Clin Microbiol.

[CR46] Stumpf AN, Roggenkamp A, Hoffmann H (2005). Specificity of enterobacterial repetitive intergenic consensus and repetitive extragenic palindromic polymerase chain reaction for the detection of clonality within the *Enterobacter cloacae* complex. Diag Microbiol Inf Dis.

[CR47] Tchounwou Paul B., Yedjou Clement G., Patlolla Anita K., Sutton Dwayne J. (2012). Heavy Metal Toxicity and the Environment. Experientia Supplementum.

[CR48] Terefework Z, Kaijalainen S, Lindström K (2001). AFLP fingerprinting as a tool to study the genetic diversity of *Rhizobium galegae* isolated from *Galega orientalis* and *Galega officinalis*. J Biotechnol.

[CR49] Versalovic J, Koeuth T, Lupski JR (1991). Distribution of repetitive DNA sequences in eubacteria and application to fingerprinting of bacterial genomes. Nucl Acids Res.

[CR50] Versalovic J, de Bruijn FJ, Lupski JR (1998). Repetitive sequence-based PCR (rep-PCR) DNA fingerprinting of bacterial genomes. bacterial genomes.

[CR51] Vincent JM (1970). A manual for the practical study of root nodule bacteria. International Biological Programme Handbook No. 15.

[CR52] Wang YP, Shi JY, Wang H, Lin Q, Chen XC, Chen YX (2007). The influence of soil heavy metals pollution on soil microbial biomass, enzyme activity, and community composition near a copper smelter. Ecotoxicol Environ Saf.

[CR53] Wani PA, Khan MS, Zaidi A (2007). Effect of metal tolerant plant growth promoting *Bradyrhizobium* sp. (Vigna) on growth, symbiosis, seed yield and metal uptake by greengram plants. Chemosphere.

[CR54] Wilson LA, Sharp PM (2006). Enterobacterial repetitive intergenic consensus (ERIC) sequences in *Escherichia coli*: evolution and implications for ERIC-PCR. Mol Biol Evol.

[CR55] Witter E, Gong P, Bääth E, Marstorp H (2000). A study of the structure and metal tolerance of the soil microbial community six years after cessation of sewage sludge applications. Environ Toxicol Chem.

[CR56] Yang J-L, Cheng A-Ch, Wang M-S, Pan K-Ch, Luo Q-H, Zhu D-K, Chen X-Y, Qi X-F (2009). New strategies for electrophoresis analysis of enterobacterial repetitive intergenic consensus PCR in animal intestinal microflora. J Microbiol Meth.

