# Supplementary material for: Genomic polymorphism of Trifolium repens root nodule symbionts from heavy metal-abundant 100-year-old waste heap in southern Poland
Source: Arch Microbiol. 2019 Jul 25;201(10):1405–14. doi: 10.1007/s00203-019-01708-x (PMC6817745; doi:10.1007/s00203-019-01708-x)
Supplement: Supplementary file 2 — Supplementary material 2 (DOC 27 kb) [file 203_2019_1708_MOESM2_ESM.doc]

Fig. S2 Dendrogram showing the genome diversity of *Trifolium repens* nodule microsymbionts obtained from Bolesław waste heap (H), and Bolestraszyce control area (K), based on the REP-PCR DNA patterns. Cluster analysis was performed by the UPGMA method. The scale at the top of the dendrogram presents the bacterial genome similarity rate (%) of 44 studied strains
