# Supplementary material for: Genomic polymorphism of Trifolium repens root nodule symbionts from heavy metal-abundant 100-year-old waste heap in southern Poland
Source: Arch Microbiol. 2019 Jul 25;201(10):1405–14. doi: 10.1007/s00203-019-01708-x (PMC6817745; doi:10.1007/s00203-019-01708-x)
Supplement: Supplementary file 3 — Supplementary material 3 (DOCX 14 kb) [file 203_2019_1708_MOESM3_ESM.docx]

Table S1 The number of identified genotypes, genotype diversity indices (*h*), Shannon’s diversity indices (*H'*), indices of strain diversity (ISD), fixation indices (F_ST_), and Simpson’s indices of diversity (*D*) of *R. leguminosarum* bv. *trifolii* isolated from nodules of *T. repens* growing in the Bolesław waste heap (metalliferous) and control (nonmetalliferous) area, determined by ERIC- PCR and REP-PCR techniques

|  | ERIC-PCR | | REP-PCR | |
| --- | --- | --- | --- | --- |
|  | control area | waste heap area | control area | waste heap area |
| No. of genotypes | 34 | 13 | 25 | 19 |
| *h* | 0.99±0.01 | 0.89±0.03 | 0.98±0.02 | 0.90±0.02 |
| *H'* | 3.4212 | 2.2632* | 3.0734 | 2.6547* |
| ISD | 61% | | 57% | |
| F_ST_^#^ | 0.162, *p*=0.008 | | 0.170, *p*=0.000 | |
| *D* | 0.9737 | | 0.9826 | |

Abbreviations

^*^ U Mann-Whitney statistical significances of differences in genotype richness (*H')* between metalliferous and nonmetalliferous populations determined by ERIC- and REP-PCR methods, (*p*<0.05)

^#^ F_ST_<0.05 indicates a little genetic differentiation, 0.15>F_ST_>0.05 – moderate genetic differentiation, 0.25>F_ST_>0.15 – great genetic differentiation, F_ST_>0.25 – very great genetic differentiation
